# Alcohol use in South Sudan in relation to social factors, mental distress and traumatic events

**DOI:** 10.1186/s12889-016-3605-9

**Published:** 2016-09-06

**Authors:** Lars Lien, Edvard Hauff, Priscilla Martinez, Arne H. Eide, Leslie Swarts, Touraj Ayazi

**Affiliations:** 1 Department of Public Health, Hedmark University College, Elverum, Norway; 2 National Advisory Board for Dual Diagnosis, Hospital Innlandet Trust, Box 104, 2381 Brumunddal, Norway; 3 Department of Mental Health and Addiction, University of Oslo, Oslo, Norway; 4 Norwegian Center for Addiction Research, University of Oslo, Oslo, Norway; 5 SINTEF Technology and Society, Oslo, Norway; 6 Department of Psychology, Alan J. Flisher Centre for Public Mental Health, Stellenbosch University, Matieland, South Africa

**Keywords:** Alcohol, AUDIT, Distress, Poverty, Trauma

## Abstract

**Background:**

Alcohol use is a major public health problem with vast implications for poor, war-torn countries. The objective of this study was to describe prevalence of alcohol use and risky drinking across socio-demographic factors in South Sudan, and to determine the association between risky drinking, traumatic events and mental distress.

**Methods:**

This is a randomized, population based, cross-sectional study from the north-western part of South Sudan with nearly 500 participants. We used the Alcohol Use Disorders Identification Test (AUDIT) as main outcome variable, the General Health Questionnaire (GHQ-28) for mental distress and five questions to assess traumatic events.

**Results:**

The mean AUDIT score was 2.7 (SD 0.3) with 14,2 % in the high risk problem drinking category. Being male, lack of a regular income and psychological distress were significantly associated with higher AUDIT score. Traumatic events, however, was not associated with higher score on AUDIT.

**Conclusion:**

Despite decades of civil war and great poverty the alcohol use in this population was at the same level as other countries in Southern Africa. Traumatic events were not related to risk of problem drinking.

## Background

Alcohol use and risky drinking are critical public health issues worldwide, evidenced by alcohol as the third highest contributor to the global burden of disease [1]. Countries experiencing war, poverty and conflict are at particular risk for the negative health impacts of alcohol use, given the chaos and insecurity of war-afflicted environments and the lack of resources to address the negative health and social consequences of risky drinking [2]. Moreover, exposure to war, loss, violence, and forced displacement can increase the risk of psychological distress, including symptoms of depression, anxiety, and post-traumatic stress disorder, which may lead to and exacerbate the effects of harmful alcohol use [3].

South Sudan is one of the most economically disadvantaged countries in the world [4], and has experienced more than 20 years of armed conflict. The signing of the Comprehensive Peace Agreement in 2005 ended extensive war-related violence and large-scale forced displacement, and resulted in the creation of the new state of South Sudan in 2011. Despite this positive change, the growing influx of returnees to South Sudan has placed an extraordinary strain on already scant services and resources. In addition, violent intertribal conflict, although not a new phenomenon, took on a new and dangerously politicized character in recent years [5]. This contemporary violent history has resulted in high rates of trauma among the general population of South Sudan. A cross-sectional survey among adult residents of the capital of South Sudan, Juba, observed that over 90 % of respondents had ever experienced one or more traumatic events, 51.5 % of the respondents had ever directly experienced a combat situation, and 49.6 % had witnessed the murder of family or friends [6]. Our own previous work among a general adult population sample of the Greater Bahr el Ghazal region of South Sudan in 2010 showed over a quarter of participants had experienced 8 or more traumatic events [7].

Alcohol has historically been consumed in South Sudan in cultural and religious ceremonies and also as a conduit for religious and political expression mainly for men. Local alcoholic drinks called Soko, Aragi, and Mawher, distilled from dates and yeast, are widely produced and consumed. In addition, relatively inexpensive alcoholic drinks imported from neighboring countries are available. Many women, and especially in female-headed households, are engaged in production and sale of locally produced alcohol. No treatment resources are available for alcohol and substance abuse problems [4, 6].

The association between exposure to traumatic events and experiencing mental health problems is well-established in post-conflict populations [8–10]. Few studies have been conducted among the South Sudanese population, although these studies expectedly observed significant associations between trauma exposure and mental health problems [6, 11, 12]. Risky alcohol use is also a potential serious negative consequence of exposure to traumatic events [13], although less well-studied than other mental health problems.

Risky drinking is a concern among conflict-affected populations in resource poor countries for several reasons. First, both exposure to traumatic events and subsequent mental health problems are each associated with alcohol use problems, with alcohol used as a form of self-medication to alleviate symptoms of these disorders [14–16]. Second, alcohol may be used as a coping strategy to manage the impoverishment, displacement and loss often inflicted by armed and ongoing conflict [17–19]. Third, risky drinking is causally associated with several non-communicable diseases (NCDs), such as cardiovascular disease, cirrhosis, alcohol hepatitis, and diabetes, which are of growing significance among conflict-affected populations [20, 21]. Fourth, risky drinking is an important contributor to gender-based violence in the form of rape of women, which is a major concern in many settings affected by armed conflicts [22–24].

To the best of our knowledge, the relationship between trauma, alcohol use and risky drinking has not been investigated in South Sudan. Indeed, given the recency of the creation of South Sudan as a nation state, there are no data on alcohol consumption levels produced by the local government, nor were South Sudan included in the latest Global Report on Alcohol use by the World Health Organization [25]. However, local media reports suggest high levels of risky alcohol use and growing public concern over the negative health and social impacts of increasing alcohol use [26–28]. The lack of empirical data on the prevalence of alcohol use within the context of South Sudan’s serious and recent traumatic history may result in people with alcohol use problems and concomitant traumatic backgrounds and mental distress going unidentified and untreated. Information about the levels of alcohol use and associated demographic factors, and how alcohol use is associated with trauma would aid health planners and providers to assess the extent and nature of alcohol use in South Sudan, and plan mental health services accordingly. There is reasons to believe that as alcohol use and risky drinking are critical public health issues worldwide, evidenced by alcohol as the third highest contributor to the global burden of disease [1] this would probably also count for South-Sudan.

This study aimed to describe the prevalence of alcohol use and risky drinking across socio-demographic factors in a community-based population exposed to high levels of war-related trauma in South Sudan, and to determine the association between risky drinking and traumatic events controlling for mental distress.

## Method

We conducted a cross-sectional community survey in the Greater Bahr el Ghazal States, South Sudan in 2012. A multistage random cluster sampling method was used. Eight randomly selected administrative units (‘Boma’) constituted the survey clusters. The population size was obtained by adding up the population of each Boma.

Boma consists of both rural and urban areas which is adapted from the local authorities’ classification of the areas. However the urban areas may be characterized as semi-urban; they include a mixture of both rural and urban properties, and are much less developed than the larger, more cosmopolitan African cities like Nairobi, Abuja, and Kampala. The population data were based on the 2008 Sudan census [29]. These data were considered the most accurate population data available. In the next stage, the ‘spin-the-pen’ method from the WHO Expanded Programme on Immunization [30] was used for household selection: the approximate geographic centre of the area was identified and one household along an imaginary line connecting the centre to the periphery was selected at random. Subsequent households were then selected by visiting every third-closest household. Within each selected household, individuals who were 18 years or older and gave informed consent to take part in the study were assigned a number. A card was drawn at random from a deck of cards with corresponding numbers, and the household member with that number was then interviewed.

The interviewers were health personnel (three women and three men) from the region who were familiar with the cultural traditions and fluent in the relevant local languages. They participated in training workshops (3 days) prior to the data collection, during which they were trained in using the survey instruments, and the cultural acceptability of the interview protocol was discussed. The training was conducted by LL, EH, and TA. The research instruments were available in both English and Arabic, but the main language used was Arabic. In addition, the key terms of the questionnaire were discussed and translated into the indigenous languages of the area to ensure that the interviewers could easily explain all the items to the participants. Each household was approached by both a male and a female interviewer to ensure the interviewer’s gender would match that of the participant. Ethical clearance was obtained from the Research Department in the Ministry of Health of the Government of South Sudan and the Norwegian Regional Committee for Medical Research.

### Instruments

A questionnaire designed to gather information about socio-demographic factors was administered to all participants which included gender, age, urban/rural setting, marital status, level of education, employment status and income.

We used the Alcohol Use Disorders Identification Test (AUDIT) to identify people with past year alcohol use and risky drinking patterns [31]. The AUDIT is a ten-item assessment developed by the World Health Organization to identify people with risky drinking patterns. Questions address alcohol consumption, alcohol dependence symptoms (according to DSM-IV criteria), and alcohol-related problems. Item response scoring ranges from 0 to 4 and we produced a total score as the sum of all item responses, where higher scores indicate a higher likelihood of an risky alcohol drinking. We created a trichotomous indicator variable for drinking pattern with the categories of “not current drinkers”, “low risk drinkers” and “harmful/hazardous drinkers”. We labelled people who reported no alcohol use in the last 12 months as “not current drinkers”. We used the WHO recommended cut-off score of 8, and categorized people with a score below 8 as “low risk drinkers”, and those with a score of 8 or greater as “harmful/hazardous drinkers” [31].

To assess exposure to recent traumatic events, participants were asked whether (1) their property had been looted, confiscated or destroyed; (2) they had been exposed to a combat situation; (3) they had suffered serious physical injuries; (4) their family members had experienced serious physical injures; and (5) they had experienced the disappearance or kidnapping of a family member. Responses were recorded as ‘yes’ or ‘no’ to each event. We included these items because they have frequently been reported in recent studies conducted in the same region of South Sudan [6, 7]. We calculated a total trauma exposure score by summing the responses of the five types of traumatic events (range = 0–5), with higher scores representing a higher level of exposure to traumatic events.

We measured psychological distress using the General Health Questionnaire (GHQ-28). The GHQ-28 is a screening instrument that is widely used to detect psychological distress in community settings and non-psychiatric clinical settings [32]. It has been used in various populations and cultural settings, including Sudan [12]. Each item has a four-point severity scale (‘not at all’, ‘no more than usual’, ‘rather more than usual’ and ‘much more than usual’) with corresponding values of 0, 1, 2 or 3. We calculated a total GHQ-28 score for each participant by adding the scores for each individual item. A higher total score on the GHQ-28 indicates more severe psychological distress (score range = 0–84).

Internal reliability was evaluated using Cronbach’s alpha. It was estimated to be 0.94 for GHQ-28 (psychological distress), 0.76 for the traumatic events assessment. The obtained Cronbach’s alpha values were above the commonly accepted level of 0.70.

### Data analyses

Data analyses were conducted using SPSS (PASW) version 20.0. Missing data were excluded from the analysis. For any given variable, the maximum amount of missing data was less than 5 %. Descriptive statistics are presented as frequencies and means. For bivariate comparisons we used chi-square tests of independence for categorical variables, and ANOVA for continuous measures across the 3 drinking categories. We used multinomial regression analysis with the trichotomous drinking variable as the dependent variable, controlling for gender, age, urban/rural setting, marital status, level of education, employment status, having a regular monthly income, exposure to traumatic events, and psychological distress.

## Results

Sample characteristics are presented in Table 1. Overall, a total of 465 people were included in the survey. The sample was comprised of 53.2 % women, 52.5 % people living in urban settings, and 73.7 % married individuals. Over a third of the participants did not have a regular income and over 40 % did not have any educational background. The average number of negative life events was 1.5 (SD 1.7), the mean AUDIT score was 2.7 (SD 6.8) and the mean GHQ was 23.2 (SD 14.7).

**Table 1:** Characteristics of participants

Variable	N (%)
Sex	
Male	217 (46.8)
Female	247 (53.2)
Urban/rural setting	
Urban	243 (52.5)
Rural	220 (47.5)
Age (years)	
18–25	132 (28.5)
26–35	185 (39.8)
36–50	113 (24.4)
> 50	33 (7.1)
Marital status	
Single	120 (26.3)
Married	336 (73.7)
Education	
Secondary school or higher	123 (26.6)
Primary school	134 (28.9)
Did not attend school	206 (44.5)
Employment	
Paid work	333 (76.9)
Student	57 (13.2)
Unemployed	43 (9.9)
Regular monthly income	
No	177 (38.6)
Yes	282 (61.4)
Exposure to recent traumatic event	
Property looted, confiscated or destroyed	205 (44.3)
Exposed to combat situation	96 (20.8)
Serious physical injuries	80 (17.3)
Serious physical injuries of family members	192 (41.5)
Disappearance or kidnapping of a family member	101 (21.8)
	Mean (95 % CI)
Traumatic events	1.45 (1.31–1.60)
Psychological distress^a^	23.2 (21.8–24.5)

^a^Psychological distress: General Health Questionnaire (GHQ-28)

Selected sociodemographics by drinking status are presented in Table 2. We identified 329 (70.8 %) people who reported no alcohol use in the last 12 months, 70 (15.1 %) who reported low risk drinking, and 66 (14.2 %) who reported harmful/hazardous drinking, A greater proportion of both low risk and harmful/hazardous drinkers are in the age groups above 25 years old compared to people not currently drinking. Being unemployed was highest among harmful/hazardous drinkers relative to both not currently and low risk drinking individuals (28.6 % vs. 7.7 % and 4.7 %, *p* < 0.01), respectively). Both the mean level of psychological distress (32.7, SE 1.84) and exposure to traumatic events (2.4, SE 0.18) was highest among people with harmful/hazardous drinking.

**Table 2:** Selected sociodemographic characteristics by drinking status among community dwelling adults in South Sudan

	No current drinking	Low risk drinking	Harmful/hazardous drinking	*p*-value
	*n* = 329	*n* = 70	*n* = 66	
	n (%)			
Gender				
Female	184 (55.9)	33 (47.8)	30 (45.5)	0.19
Male	145 (44.1)	36 (52.2)	36 (54.5)	
Age				
18–25	106 (32.2)	11 (16.2)	15 (22.7)	0.002
26–35	138 (42.0)	23 (33.8)	24 (36.4)	
36–50	67 (20.4)	27 (39.7)	19 (28.8)	
> 50	18 (4.4)	7 (10.3)	8 (12.1)	
Setting				
Urban	160 (48.6)	46 (67.7)	37 (56.1)	0.014
Rural	169 (51.4)	22 (32.3)	29 (43.9)	
Marital status				
Single	89 (27.4)	16 (24.2)	15 (23.1)	0.71
Married	236 (72.6)	50 (75.8)	50 (76.9)	
Education				
No formal education	146 (44.4)	26 (38.2)	34 (51.6)	0.340
Primary school	100 (30.4)	18 (26.5)	16 (24.2)	
Secondary or higher	83 (25.2)	24 (35.3)	16 (24.2)	
Employment				
Employed	246 (78.6)	51 (79.7)	36 (64.3)	0.001
Student	43 (13.7)	10 (15.6)	4 (7.1)	
Unemployed	24 (7.7)	3 (4.7)	16 (28.6)	
Regular monthly income				
Yes	204 (62.4)	49 (73.1)	29 (44.6)	0.003
No	123 (37.6)	18 (26.9)	36 (55.4)	
Psychological distress				
(mean, SE)	20.8 (0.78)	21.2 (1.71)	32.7 (1.84)	0.001
Traumatic events				
(mean, SE)	1.5 (0.09)	0.74 (0.16)	2.4 (0.18)	0.001

Note: Not all frequency sums across sociodemographics are equal due to missing data

Results from the multinomial regression are presented in Table 3. In adjusted analysis, relative to people not currently drinking, low risk drinking was associated with being above 25 years of age, a higher level of psychological distress and lower exposure to traumatic events. For harmful/hazardous drinking, being male and having greater psychological distress increased the risk of harmful/hazardous drinking compared to not currently drinking.

**Table 3:** Results for multinomial regressions of low risk and harmful/hazardous drinking relative to people not currently drinking among community dwelling adults in South Sudan

	Low risk drinking	Harmful/hazardous drinking
	Adjusted ORs (95 % CI)	
Gender		
Female	1	1
Male	1.40 (0.73,2.26)	3.10 (1.46,6.45)**
Age		
18–25	1	1
26–35	2.83 (1.0,8.0)*	1.20 (0.46,3.14)
36–50	5.86 (1.96,17.53)**	2.25 (0.80,6.30)
> 50	5.56 (1.36,22.79)*	1.61 (0.41,6.27)
Setting		
Rural	1	1
Urban	1.71 (0.81,3.63)	1.12 (0.51,2.44)
Marital status		
Single	1	1
Married	1.62 (0.68,3.85)	1.31 (0.50,3.45)
Education		
No formal education	1	1
Primary school	1.07 (0.48,2.38)	0.88 (0.37,2.06)
Secondary or higher	1.42 (0.62,3.25)	1.16 (0.46,2.94)
Employment		
Employed	1	1
Student	3.21 (0.91,11.29)	0.37 (0.07,1.92)
Unemployed	0.78 (0.14,4.28)	2.20 (0.74,6.50)
Regular monthly income		
Yes	1	1
No	0.86 (0.37,2.00)	1.88 (0.81,4.38)
Psychological distress (mean, CI)	1.01 (0.98,1.03)**	1.04 (1.01,1.06)**
Traumatic events (mean, CI)	0.65 (0.49,0.88)**	1.18 (0.95,1.48)

*p < 0.05, ** *p*-value < 0.01

## Discussion

The main finding from this study was that just over 14 % of the participants were identified as harmful/hazardous drinkers based on the AUDIT. Being male, lack of regular income and psychological distress were the main risk factors for alcohol misuse among this randomly selected population from north western part of South Sudan. This is in line with a study on drinking patterns in 20 different African countries which found that middle-aged, adult sub-Saharan African males appeared to be the most common alcohol consumers as well as risky drinkers in Africa [33].

It is difficult to compare prevalence of high risk drinking between our study and the WHO study referred above due to use of different measures, but our figures are in the low end as the prevalence varied between 7 and 77 % between the countries [33]. Neither Sudan nor South Sudan was part of the study. In another study from the same WHO material Rossow and Clausen claim that the distribution patterns of alcohol consumption in African countries are consistent with those observed previously in industrialized countries which support the universality of the observation that the prevalence of risky drinking is linked closely to mean consumption [34].

As found in our study, numerous investigations also from African countries have shown that there is a strong association between mental health problems and alcohol misuse [35, 36]. Several hypotheses for this relation have been proposed and the one with the strongest evidence currently suggests a causal linkage between alcohol use disorders and major depression, such that increasing involvement with alcohol increases risk of depression [37].

Although a greater percentage of harmful/hazardous drinkers had no regular monthly income compared to those currently not drinking and low-risk drinkers, regular monthly income did not differentiate harmful/hazardous drinkers from those who did not currently drink in the multinomial regression analysis. . This has been shown in longitudinal studies from large cohort studies in western countries [38] and in African studies related to unemployment [39]. One unexpected finding from this study was that traumatic events were not associated with alcohol misuse when controlling for other factors. This is contrary to other studies showing that post-traumatic stress is strongly linked to alcohol misuse [35] and might be due to competing risk from other factors in our study like poverty.

As already mentioned and despite our lack of association between alcohol use and traumatic events we would reiterate that countries experiencing war and poverty are prone to the impacts of alcohol use [2] and that exposure to war and forced displacement might increase the risk of psychological distress, including post-traumatic stress disorder, which might exacerbate the harmful effects of alcohol use [3]. One implication from this study is therefore to make efforts to reduce the devastating effects of alcohol use in resource poor and war affected areas by putting restrictive measures in place like tax and availability. To join forces with opinion leaders like chiefs and priests to have then to talk about the negative effects of alcohol might also help reducing consumption.

### Limitation

The current study had some limitations. Being a cross-sectional study, it cannot identify cause-and-effect relationships between various independent variables and health outcomes. The 2008 Sudan census, which was used as the source of population data and in the sampling process, has inaccuracies, particularly because of the large-scale migration process and the influx of returnees. In addition, the a priori exclusion of the insecure areas creates a bias that is difficult to estimate. These limitations influence the generalizability of our findings. The use of an additive scale of traumatic events is a simple way of including an indicator of exposure. However, this would not differentiate between the types and severity of events. It is also a limitation that we lack information of time since trauma. The method of selecting participants from each household may potentially cause a bias if the selected participant is absent. In the current study however, all the selected interviewees were traced and no absentee was reported.

## Conclusions

We were not able to show any relationship between alcohol use and neither current nor past traumatic events in a population suffering from long standing civil war and great poverty. The use of alcohol was at the same level as the rest of Southern Africa. Problematic alcohol use was related to male sex, lack of income and recent psychological distress.
